# Mechanistic Insights into the Structural Evolution of ZIF‐67 via Electrospinning Strategy Toward High Electromagnetic Wave Absorption Performance of ZIF‐67‐Derived Carbon Nanofibers

**DOI:** 10.1002/advs.202502560

**Published:** 2025-04-07

**Authors:** Xinhui Cao, Xinyi Wu, Xue Wang, Jiamei Luo, Zhe Zhang, Yi Xue, Guoliang Zhang, Liying Zhang, Hui Zhang, Jianyong Yu

**Affiliations:** ^1^ Shanghai Collaborative Innovation Center of High‐Performance Fibers and Composites (Province‐Ministry Joint), Center for Civil Aviation Composites, College of Materials Science and Engineering, Donghua University Shanghai 201620 P. R. China; ^2^ Center for Computational Chemistry, College of Chemistry and Chemical Engineering Wuhan Textile University Wuhan 430200 P. R. China; ^3^ College of Textiles Donghua University Shanghai 201620 P. R. China; ^4^ Shanghai Collaborative Innovation Center of High‐Performance Fibers and Composites (Province‐Ministry Joint), Center for Civil Aviation Composites Donghua University Shanghai 201620 P. R. China

**Keywords:** carbon nanofibers, cobalt‐based zeolitic imidazolate framework, electromagnetic wave absorption, ex situ electrospinning, in situ electrospinning

## Abstract

Metal‐organic framework (MOF)‐derived architectures are regarded as an effective electromagnetic wave (EMW)‐absorbing materials owing to their adjustable compositions and microstructures. The combination of MOFs with carbon nanofibers (CNFs) is a practical method to increase the EMW absorption ability. In this work, cobalt‐based zeolitic imidazolate framework‐67 (ZIF‐67) serves as a self‐sacrificing precursor to fabricate Co‐carbon nanofiber (Co‐CNF) composites via an in situ electrospinning strategy. Comparative studies on ex situ and in situ, electrospinning strategies for EMW absorption are conducted. A unique structural evolution mechanism from ZIF‐67 to Co nanoparticles is explored. Numerous small Co nanoparticles are evenly distributed on the surface of in situ synthesized Co‐CNF (in‐Co‐CNF) resulting from the collapse of the ZIF‐67 framework, whereas the ZIF‐67 framework remains on the surface of ex situ synthesized Co‐CNF (ex‐Co‐CNF), encapsulating large Co nanoparticles. A lower reflection loss (*RL*) of −48.6 dB at 6.8 GHz with 3.5 mm is achieved for the in‐Co‐CNF because of the improved conduction, polarization, and magnetic losses, whereas the ex‐Co‐CNF only exhibits an *RL* of −18.3 dB at 9.3 GHz with the same thickness. A radar cross‐section (RCS) simulation and a Tesla wireless transmission experiment are conducted to validate the EMW absorption of Co‐CNF composites in real applications.

## Graphical abstract

With 5G and AI robotics rising, electromagnetic radiation risks signal security and health. This work investigates the electromagnetic wave absorption of Co/CNF composites via in situ and ex situ electrospinning strategies. The in situ synthesized sample exhibits superior absorption properties due to enhanced structural uniformity, improved Co distribution, and optimized impedance matching, achieving a minimum *RL* of −48.6 dB at 6.9 GHz.

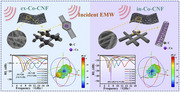

## 1 Introduction

The swift advancement of 5G technology is propelling humanity into an era dominated by intelligent AI robotics. Nevertheless, excessive exposure to electromagnetic (EM) radiation, particularly in the microwave spectrum, poses risks to signal transmission security and human health.^[^ ^1^, ^2^, ^3^ ^]^ To mitigate EM pollution, materials capable of absorbing and dissipating electromagnetic waves (EMW) have emerged as vital for sustainable development.^[^ ^4^, ^5^ ^]^

Among various candidate materials, carbon‐based materials such as carbon nanotubes (CNTs),^[^ ^6^ ^]^ graphene,^[^ ^7^ ^]^ and carbon nanofibers (CNFs)^[^ ^8^ ^]^ have emerged as promising candidates for EMW absorption because of their advantages of light weight, high electrical conductivity, and excellent chemical stability.^[^ ^9^, ^10^, ^11^ ^]^ However, single‐component carbon materials often face limitations, such as poor impedance matching and insufficient attenuation, which reduce the EMW absorption efficiency. To overcome these issues, hybridizing carbon materials with metals or metal oxides was a feasible approach for developing high‐performance EMW‐absorbing materials. Cai et al.^[^ ^12^ ^]^ uniformly grew CoNi nanoparticles on the surface of the CNTs and hollow microspheres. The obtained sample demonstrated a minimum reflection loss (*RL*_min_) of −66.9 dB and an effective absorption bandwidth (*EAB*) of 5.0 GHz. Cai et al. also fabricated VSe_2_/CNTs/Fe_3_O_4_ ternary composites^[^ ^13^ ^]^ and CNTs/Fe_3_O_4_‐carbonyl‐iron (CI) buckypapers,^[^ ^14^ ^]^ which presented *RL*_min_ values of ‐51.0 and ‐52.0 dB, respectively. Similarly, Du et al.^[^ ^15^ ^]^ synthesized FeCo/graphene nanocomposites, achieving an *RL*_min_ of −71.63 dB and an *EAB* of 4.2 GHz. The EMW absorbing materials prepared in the above works were all made by the combination of conduction loss, magnetic loss, and polarization loss components, thereby optimizing the intrinsic EM parameters. Therefore, the EMW absorption performance could be optimized through tuning the intrinsic EM parameters of the absorbers.^[^ ^16^ ^]^

Metal‐organic frameworks (MOFs) possess unique advantages, including structural tunability and extensive specific surface area, as precursors for the preparation of functional composite materials. Different MOF derivatives have been explored for EMW absorption applications through adjusting their compositions and morphologies.^[^ ^17^ ^]^ The integration of MOFs with carbon materials offers a valuable approach to improve EMW absorption performance.^[^ ^18^ ^]^ For example, Guo et al.^[^ ^19^ ^]^ prepared Ni‐MOF/CNT buckypaper with an *RL*_min_ of −57.8 dB and an *EAB* of 6.3 GHz. Luo et al.^[^ ^20^ ^]^ combined CoMn‐MOF‐74 with reduced graphene oxide (rGO) to prepare Co/MnO/porous carbon (PC)/RGO composites. The resulting composites displayed an *RL*_min_ of −50.1 dB and an *EAB* of 5.1 GHz. Although MOFs have demonstrated attractive features as microwave absorbers, most of them were produced in powder form, and their derivatives tend to aggregate, causing potential pore obstruction and loss of active sites. Additionally, aggregation can reduce the effective loading of MOFs, which weakens their EMW absorption capabilities.^[^ ^21^ ^]^

Electrospinning technology to produce continuous nanofibers with high aspect ratios, long transmission paths, and high electrical conductivity is capable of significantly improving EMW attenuation.^[^ ^22^ ^]^ Currently, MOF‐derived CNF composites are mainly prepared via direct electrospinning of a solution with pre‐synthesized MOF nanoparticles, which is referred to the ex situ electrospinning method. For instance, Chen et al.^[^ ^23^ ^]^ incorporated pre‐synthesized Fe^III^‐MOF‐5 particles into a polyacrylonitrile (PAN) polymer solution, followed by electrospinning to produce hierarchical porous Fe/Fe₃O₄‐CNF composites, which exhibited an *RL*_min_ of −39.2 dB at 15.9 GHz and an *EAB* of 4.4 GHz. Similarly, Sun and coworkers^[^ ^24^ ^]^ synthesized Co/Ni‐CNF composites from Co/Ni‐MOF derivatives, whereas Zhou et al.^[^ ^25^ ^]^ embedded Fe‐MOF into nanofibers through an electrospinning process, resulting in bead‐like Fe‐CNF composites. The Co/Ni‐CNF and Fe‐CNF composites presented *RL_min_* values of −40.1 dB at 6.3 GHz and −30.7 dB at 8.1 GHz, respectively. In our previous work, ZnO‐CNF composites were fabricated via an in situ electrospinning strategy in which MOF precursors were introduced to the electrospinning solution, followed by the initial growth of MOF nanoparticles through electrospinning.^[^ ^21^ ^]^ The obtained ZnO‐CNF composites exhibited an *RL*_min_ of −58.0 dB at 7.5 GHz, accompanied by an *EAB* of 6.5 GHz. In addition to our work, other works using an in situ electrospinning strategy were also explored. For example, Bi et al.^[^ ^26^ ^]^ prepared an electrospinning solution by mixing 2‐methylimidazole (2‐MI) with PAN. The as‐spun PAN/2‐MI was then immersed in a solution containing cobalt cations (Co^2+^). The presence of 2‐MI provided coordination sites that facilitated the growth of ZIF‐67. The obtained Co‐N‐CNF had an *RL*_min_ of ‐61.6 dB at 11.7 GHz and an *EAB* of 4.7 GHz. Similarly, Huang and coworkers^[^ ^27^ ^]^ also employed an in situ electrospinning strategy to synthesize Co_3_O_4_‐CNF with an *RL*_min_ of −57.6 dB at 9.1 GHz and an *EAB* of 6.6 GHz.

Compared with ex situ electrospinning, the strategy of in situ electrospinning provided a one‐step integrated process for the incorporation of MOF nanoparticles. However, most studies focused on introducing different metal or metal oxide nanoparticles derived from MOFs on the surface of CNFs through electrospinning and carbonization. The transformation mechanisms of MOFs during these processes and their influence on the intrinsic EM parameters of MOF‐derived CNF composites were unclear. Understanding the structural evolution of MOFs in both in situ and ex situ electrospinning processes is crucial for advancing the design of high‐performance EMW absorption materials.

Herein, this work focused on the preparation of ZIF‐67‐derived Co‐CNF composites via ex situ and in situ electrospinning strategies. By systematically comparing the in situ and ex situ electrospinning strategies, we aimed to clarify the structural evolution of ZIF‐67 and its impact on the EMW absorption performance of Co‐CNF composites. The two strategies yield nanofibers loaded with different sizes of ZIF‐67 nanoparticles. After carbonization, small Co nanoparticles were evenly distributed on the surface of in situ synthesized Co/CNF (in‐Co‐CNF), whereas large Co nanoparticles were encapsulated in the framework of ZIF‐67 on the surface of ex situ synthesized Co/CNF (ex‐Co‐CNF). The presence of small Co nanoparticles resulted in a high specific surface area, providing improved dipole polarization. The uniformly distributed Co nanoparticles on the carbon surface contributed to enhanced interfacial polarization loss and conduction loss. Accordingly, in‐Co‐CNF showed a lower *RL*_min_ of −48.6 dB at 6.8 GHz than ex‐Co‐CNF with an *RL*_min_ of −20.5 at 6.8 GHz.

## 2 Results and Discussion

**Figure 1:**
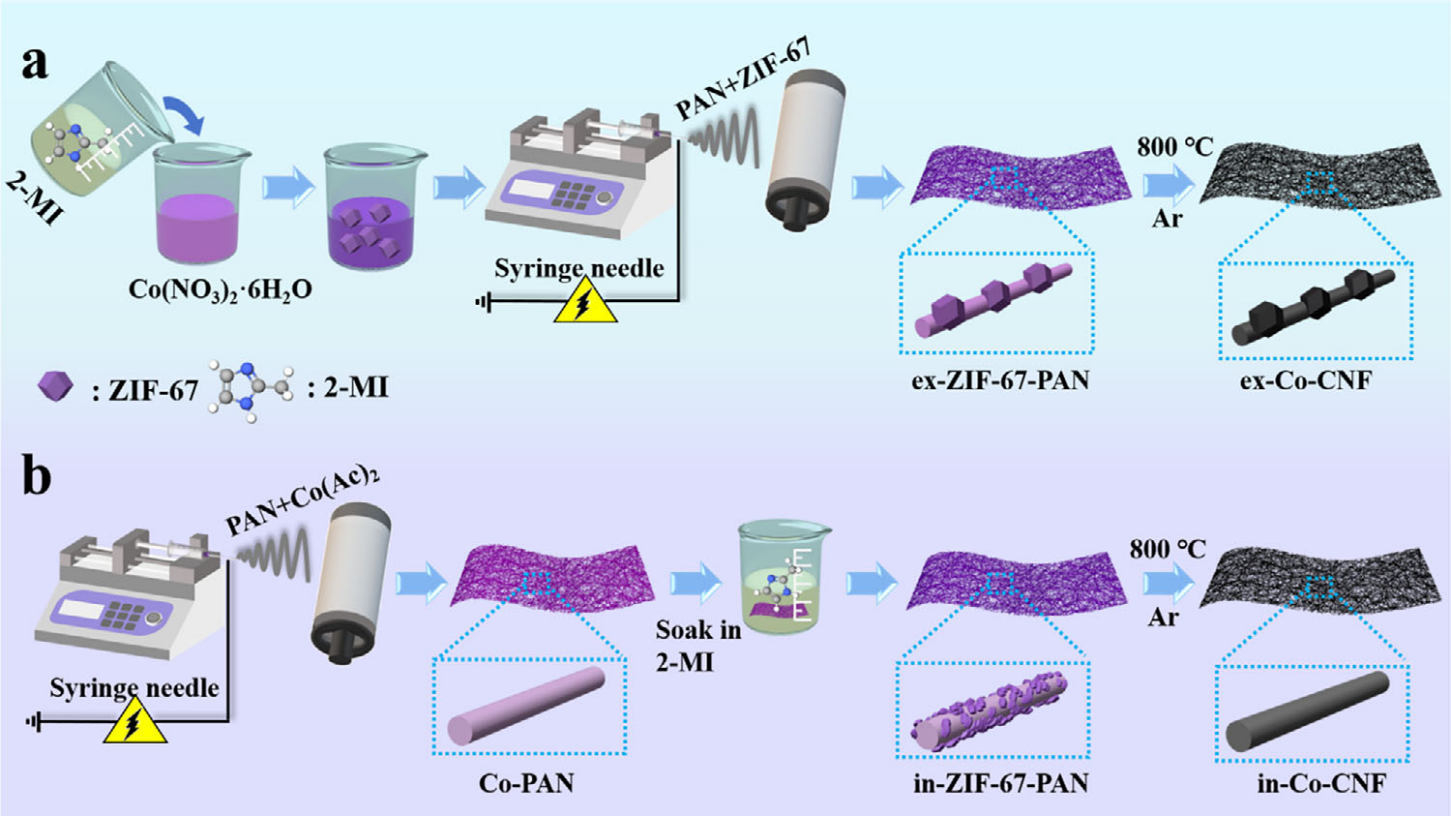
Illustration of the methods for the preparation of a) ex‐Co‐CNF and b) in‐Co‐CNF.

The synthesis process of the samples is illustrated in **Figure** **1**. In this work, 1D ZIF‐67‐derived CNF composites were fabricated via ex situ and in situ electrospinning techniques. As shown in Figure 1a, ZIF‐67 nanoparticles were synthesized from imidazolate anions and Co^2+^. The electrospinning precursor solution was prepared by mixing pre‐synthesized ZIF‐67 nanoparticles with PAN. After the ex situ electrospinning process, the ZIF‐67 nanoparticles were encapsulated in PAN to create ex‐ZIF‐67‐PAN nanofibers. In contrast, Co‐PAN nanofibers were prepared via in situ electrospinning of a precursor solution containing Co^2+^ and PAN. Following immersion in the 2‐MI/ethanol solution, Co^2+^ reacted with 2‐MI, resulting in the formation of in‐ZIF‐67‐PAN nanofibers. Finally, ex‐Co‐CNF and in‐Co‐CNF were obtained by carbonization.

**Figure 2:**
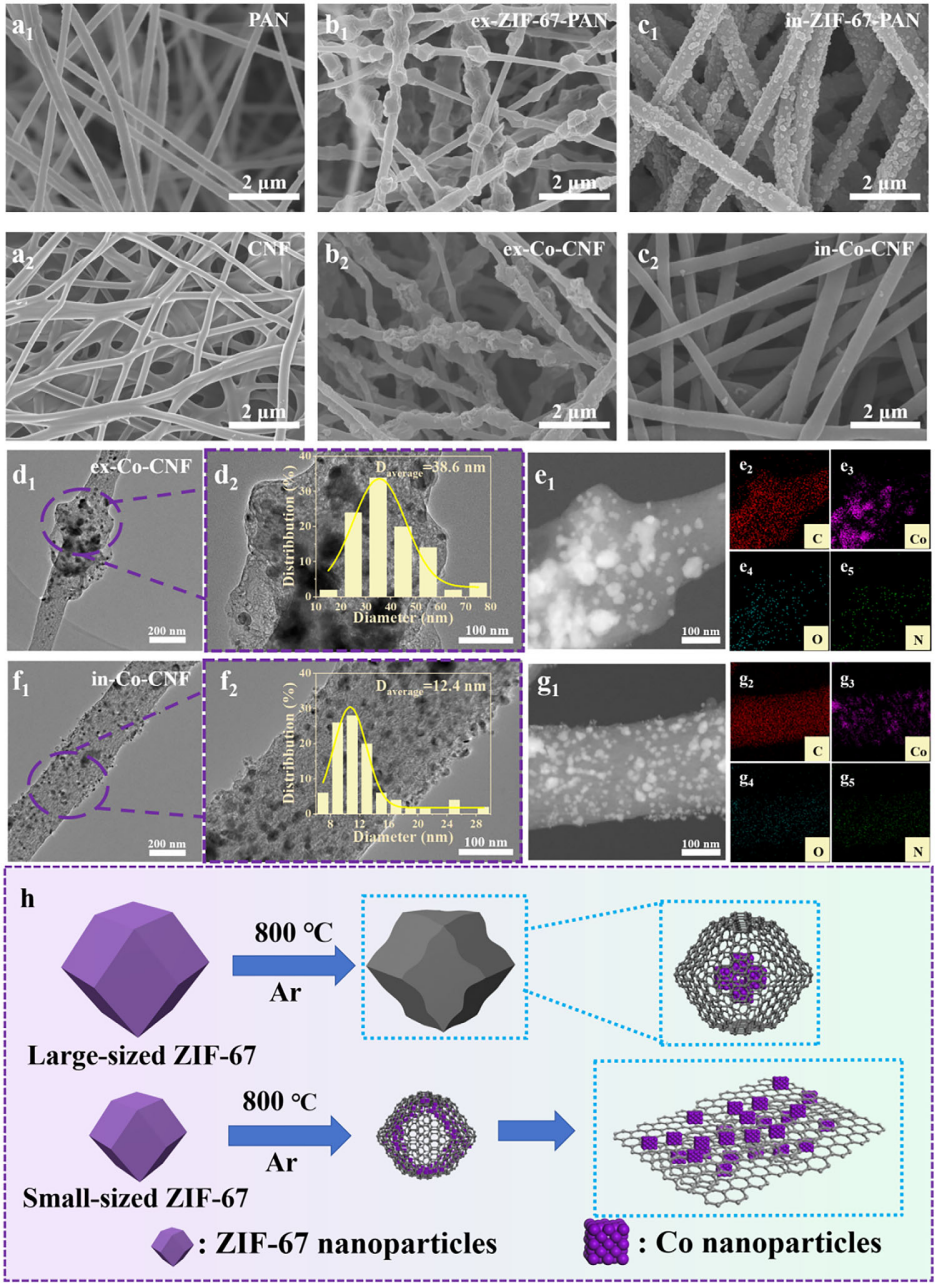
SEM images of a_1_) PAN, a_2_) CNF, b_1_) ex‐ZIF‐67‐PAN, b_2_) ex‐Co‐CNF, c_1_) in‐ZIF‐67‐PAN, and c_2_) in‐Co‐CNF; TEM images and particle size distributions of d_1_,d_2_) ex‐Co‐CNF and f_1_‐f_2_) in‐Co‐CNF; TEM images and elemental mapping of e_1_–e_5_) ex‐Co‐CNF and g_1_‐g_5_) in‐Co‐CNF; h) mechanism diagram of the transformation of ZIF‐67 into Co nanoparticles.

Figure **2**a_1_,a_2_ shows the morphologies of the PAN nanofibers and CNFs, respectively. Both the PAN nanofibers and the CNFs had diameters of 300–400 nm and relatively smooth surfaces. Figure 2b_1_ shows a unique bead‐like structure of ex‐ZIF‐67‐PAN nanofibers, in which each ZIF‐67 nanoparticle was highly isolated from each other and embedded in the PAN nanofibers. The average particle size of ZIF‐67 was ≈402 nm (Figure S1a, Supporting Information). After carbonization, the bead‐like structure remained, and slight shrinkage and wrinkling occurred on the nanoparticle surface, forming the uneven surface of the ex‐Co‐CNF (Figure 2b_2_). The morphology of in‐ZIF‐67‐PAN can be seen in Figure 2c_1_, where the nanofiber surface was uniformly decorated with ≈142 nm of ZIF‐67 nanoparticles (Figure S1b, Supporting Information). Notably, the ZIF‐67 nanoparticles disappeared on the in‐Co‐CNF surface (Figure 2c_2_), probably because of the reduction of Co^2+^ to Co nanoparticles, which caused the collapse of the ZIF‐67 framework.^[^ ^28^ ^]^ The TEM images show the distribution of Co nanoparticles in the nanofibers. The Co nanoparticles of ≈38.6 nm were mainly embedded in the polyhedral framework of ex‐Co‐CNF (Figure 2d_1_,d_2_), whereas the small Co nanoparticles of ≈12.4 nm were evenly distributed on the surface of the in‐Co‐CNF (Figure 2f_1_,f_2_). The different sizes of Co nanoparticles obtained might be attributed to the size of the ZIF‐67 nanoparticles in the PAN nanofibers before carbonization. The element mapping images in Figures 2e_1_–e_5_ and g_1_–g_5_ confirmed the even distributions of C, Co, O, and N.

To evaluate the effect of the ZIF‐67 particle size on the formation of Co nanoparticles, calculations, presented in the Appendix in the Supporting Information, were conducted. With a ZIF‐67 of ≈402.1 nm, the 39 nm Co nanoparticle contained ≈22 Co atoms. In contrast, when the size of ZIF‐67 was ≈142.4 nm, ≈34 Co atoms constituted 12 nm Co nanoparticles. Based on the above considerations, a transfer mechanism between ZIF‐67 and Co nanoparticles was proposed, as shown in Figure 2h. The coordination bond between Co^2+^ and methylimidazole was cleaved during carbonization, leading to the reduction of Co^2+^ into Co atoms. Owing to the high surface energy, self‐assembly of Co atoms occurred to form a Co nanoparticle. For large‐sized ZIF‐67, large Co nanoparticles were stabilized in the framework and thus were located far from the surface. A carbon layer was generated to encapsulate the Co nanoparticles, retaining the polyhedral morphology of the ZIF‐67 framework. In contrast, small Co nanoparticles tended to assemble near the surface. These Co nanoparticles between adjacent ZIF‐67 particles promoted the graphitization process of carbon, facilitating the reconstruction of the surface. As a result, the framework of ZIF‐67 collapsed, and the Co nanoparticles diffused on the continuous carbon networks.

**Figure 3:**
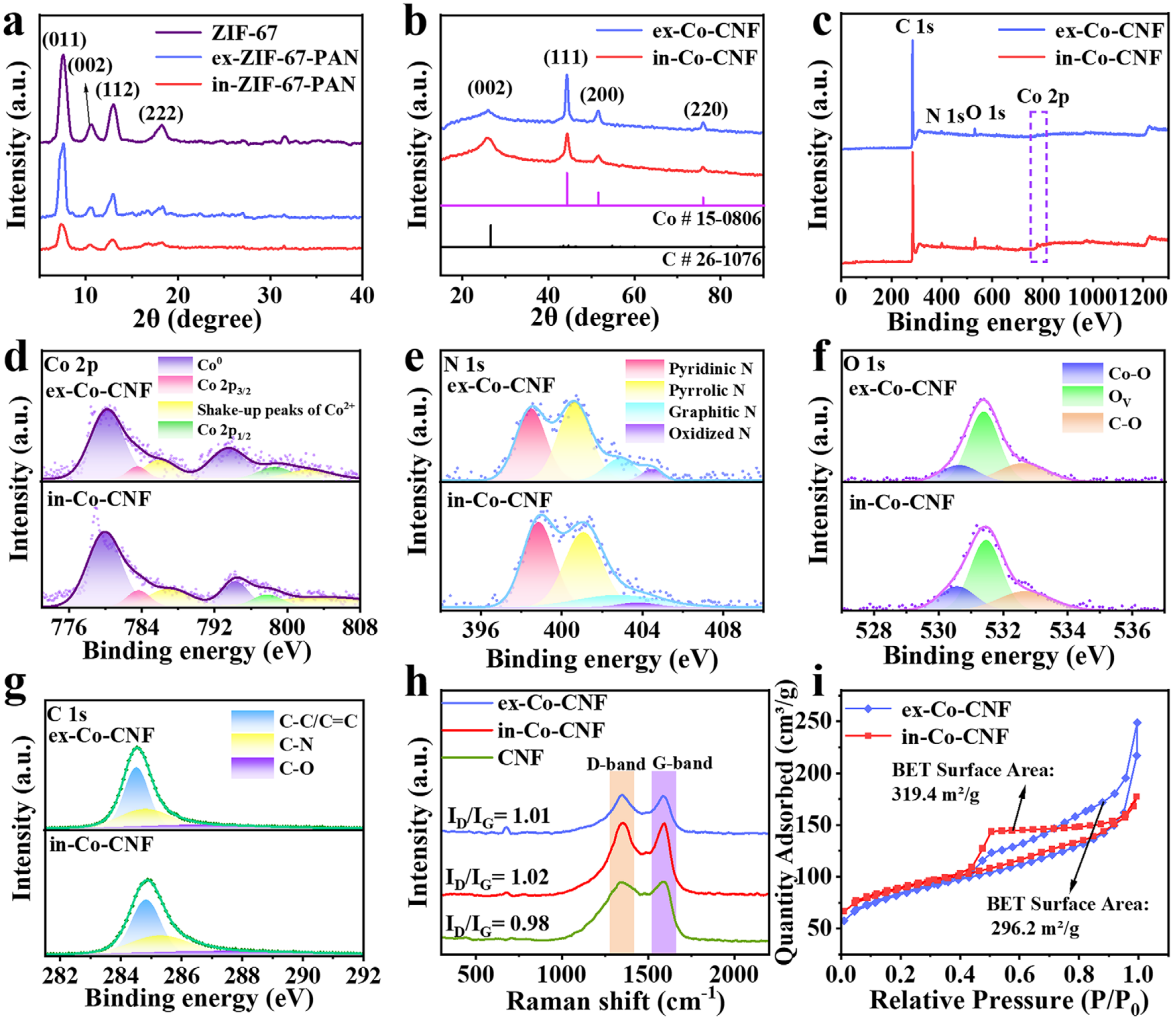
XRD patterns of a) ZIF‐67, ex‐ZIF‐67‐PAN, and in‐ZIF‐67‐PAN and b) ex‐Co‐CNF and in‐Co‐CNF; c) XPS spectra; d) Co 2p spectra; e) N 1s spectra; f) O 1s, and g) C 1s spectra of ex‐Co‐CNF and in‐Co‐CNF; h) Raman spectra of CNF, ex‐Co‐CNF and in‐Co‐CNF; i) BET information of ex‐Co‐CNF and in‐Co‐CNF.

The XRD patterns of the PAN and CNF are shown in Figure S2 (Supporting Information). PAN displayed a clear diffraction peak at 17.1°, corresponding to the (110) plane of a hexagonal structure.^[^ ^29^ ^]^ After carbonization, the peak of the carbon structures formed. **Figure** **3**a shows the XRD patterns of ZIF‐67, ex‐ZIF‐67‐PAN and in‐ZIF‐67‐PAN. ZIF‐67 exhibited peaks at 7.5, 10.5, 12.9, and 18.1°, corresponding to the (011), (002), (112), and (222) crystal planes, respectively.^[^ ^30^, ^31^ ^]^ Both ex‐ZIF‐67‐PAN and in‐ZIF‐67‐PAN exhibited the same XRD peak positions, indicating that ZIF‐67 was successfully synthesized on the PAN nanofibers.

After carbonization, the peaks observed at 44.2, 51.4, and 75.9° were assigned to the (111), (200), and (220) planes of Co,^[^ ^32^, ^33^ ^]^ respectively (Figure 3b), suggesting that Co nanoparticles formed on the CNF surface. Moreover, the Co peak intensity in the ex‐Co‐CNF sample was greater than that in the in‐Co‐CNF sample. This may be attributed to the larger size of Co particles in ex‐Co‐CNF, as larger particles and more well‐defined crystal structures typically result in higher peak intensities. Additionally, a diffraction peak appeared at 25.4°, assigned to carbon with a low degree of graphitization.^[^ ^34^ ^]^ The XPS spectra are shown in Figure 3c, revealing the presence of Co, N, O, and C on ex‐Co‐CNF and in‐Co‐CNF. In the Co 2p spectra of the in‐Co‐CNF (Figure 3d), the peaks at 779.3 and 793.2 eV (purple color) confirmed the existence of metallic Co.^[^ ^35^ ^]^ Additionally, the peak at 783.6 eV (Co 2p_3/2_, pink color) and the peak at 797.7 eV (Co 2p_1/2_, green color) were associated with two satellite peaks at 785.6 and 801.5 eV (yellow color) corresponding to Co^2+^, indicating the presence of a small amount of CoO, which was due to incomplete reduction during the carbonization process.^[^ ^36^ ^]^ Owing to the extremely low content of CoO, the influence of CoO on the overall EMW absorption performance was neglected. The high‐resolution N 1s spectra in Figure 3e exhibited peaks at 398.5, 401.1, 403.3, and 403.9 eV, which were attributed to pyridinic N, pyrrolic N, graphitic N, and oxidized N, respectively. Graphic N mainly contributed to conduction loss, whereas the other materials were responsible for enhancing dipole polarization.^[^ ^37^ ^]^ In Figure 3f, the peak at 530.4 eV was associated with Co─O, further confirming the presence of CoO.^[^ ^38^ ^]^ The peaks at 531.4 and 533.1 eV corresponded to vacancy oxygen (O_v_) and C─O, respectively. Both can act as dipole centers, causing polarization relaxation.^[^ ^39^ ^]^

Figure 3g presents the C 1s spectra. The peaks located at 284.5, 285.2, and 286.7 eV corresponded to C─C/C═C, C─N, and C─O bonds, respectively.^[^ ^40^ ^]^ Raman spectroscopy was used to analyze the disorder in the carbon lattice. All the samples exhibited two peaks at 1352.7 and 1590.5 cm^−1^, assigned to the D band (related to structural defects) and the G band (related to crystalline graphite), respectively. As shown in Figure 3h, the *I*_D_/*I*_G_ values of the CNF, ex‐Co‐CNF, and in‐Co‐CNF were 0.98, 1.01, and 1.02, respectively, indicating that there was no significant difference in the carbon lattice structures among the samples. Because the *I*_D_/*I*_G_ ratio is typically associated with the electrical conductivity of carbon materials, the electrical conductivities of the CNF, ex‐Co‐CNF, and in‐Co‐CNF were measured, as shown in Figure S3 (Supporting Information). The electrical conductivity of both ex‐Co‐CNF and in‐Co‐CNF was greater than that of CNF, which could be attributed to the introduction of Co nanoparticles during the carbonization process. To identify the difference in electrical conductivity between ex‐Co‐CNF and in‐Co‐CNF, inductively coupled plasma (ICP) measurements were conducted to confirm the content of Co. As shown in Table S1 (Supporting Information), the Co content was nearly identical in both samples. Therefore, it can be deduced that the size and distribution of Co nanoparticles played vital roles in the electrical conductivity of ex‐Co‐CNF and in‐Co‐CNF. Compared with ex‐Co‐CNF, the smaller and more evenly distributed Co nanoparticles on the surface of in‐Co‐CNF facilitated electron transfer, ultimately leading to a higher electrical conductivity. Figure 3i shows the BET test results of ex‐Co‐CNF and in‐Co‐CNT, revealing mesoporous properties with narrow pore size distributions.^[^ ^41^ ^]^ The specific surface areas of ex‐Co‐CNF and in‐Co‐CNF were 296.2 and 319.4 m^2^ g^−1^, respectively. The large surface area of the in‐Co‐CNF resulted from the smaller particle size of the Co nanoparticles, which was caused by the self‐sacrificial behavior of ZIF‐67 in the in situ electrospinning method. The increase in specific surface area contributed to the enhancement of the multiple scattering and reflection of EMW.

**Figure 4:**
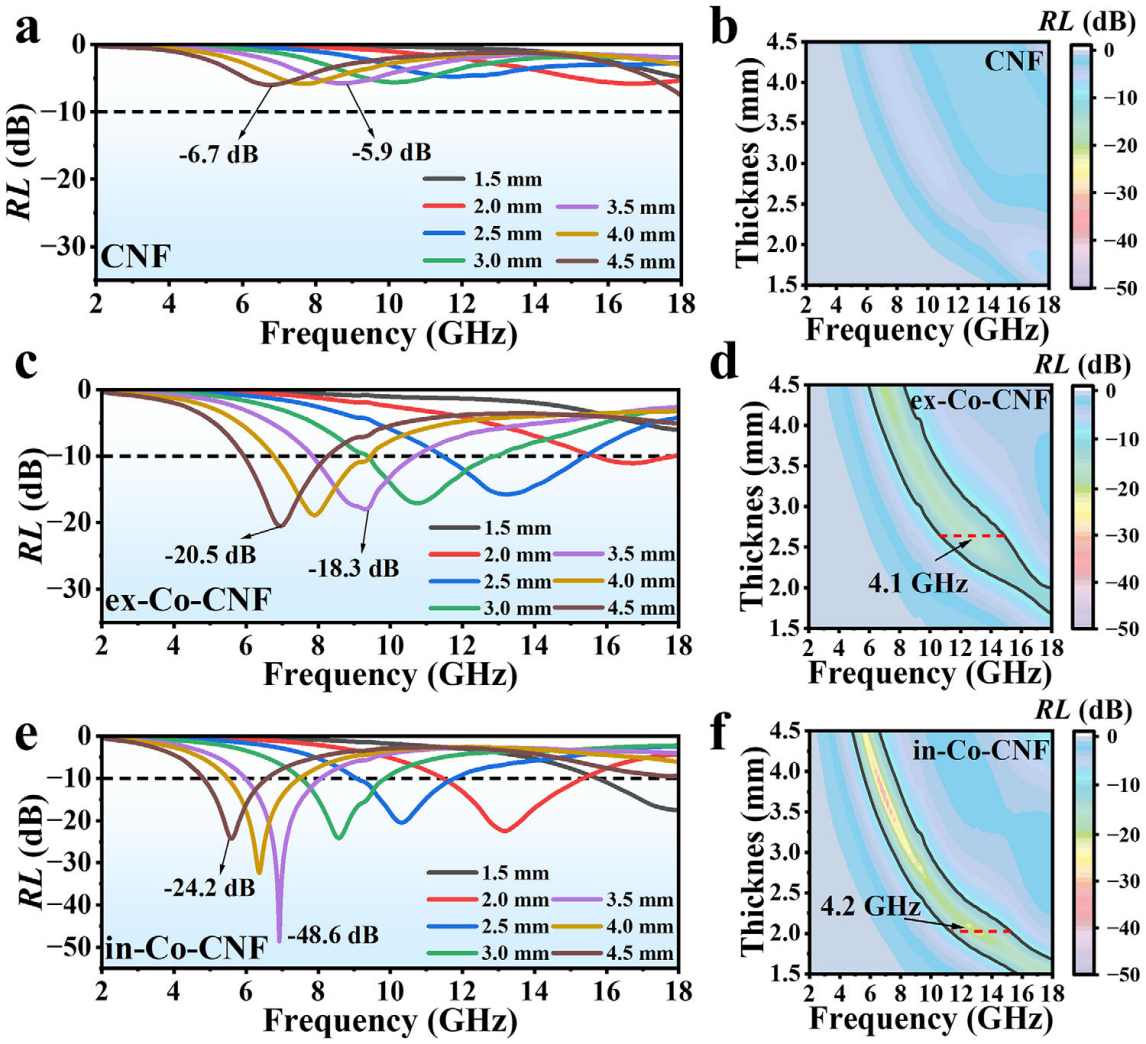
2D *RL* plots at different thicknesses and *RL* curves at selected thicknesses of a,b) CNF, c,d) ex‐Co‐CNF, and e,f) in‐Co‐CNF.

To assess the EMW absorbing capability, the *RL* was obtained via Equations (1) and (2),^[^ ^42^, ^43^ ^]^

(1) $$Z_{in}=\sqrt{\frac{\mu_{r}}{\varepsilon_{r}}}\;\tanh\left[j\left(\frac{2\pi }{c}\right)fd\;\left(\sqrt{\mu_{r}\varepsilon_{r}}\right)\right]$$

(2) $$RL=20\;\mathrm{lg}\left|\frac{Z_{in}-Z_{0}}{Z_{in}+Z_{0}}\right|$$

where *Z_in_* and *Z_0_* are the input impedance and the free‐space impedance, respectively. *f* is the frequency, *c* is the speed of light, and *d* represents the matching thickness of the absorber. *µ_r_* and ε_*r*_ denote the complex magnetic permeability and the complex permittivity, respectively. When the *RL* is ≤−10 dB, the sample is normally considered capable of absorbing 90% of the EMW, and the corresponding bandwidth represents the *EAB*.^[^ ^44^ ^]^ **Figure** **4** shows that the *RL_min_* of CNF was −6.7 dB at 6.8 GHz. In contrast, ex‐Co‐CNF and in‐Co‐CNF had superior EMW absorbing capabilities with the incorporation of Co nanoparticles. At the same thickness (3.5 mm), in‐Co‐CNF achieved a notable *RL* of −48.6 dB at 6.8 GHz compared with ex‐Co‐CNF (−18.3 dB at 9.3 GHz). Interestingly, similar *EAB* values of ≈4.2 GHz were achieved for in‐Co‐CNF and ex‐Co‐CNF, but the corresponding matching thickness of in‐Co‐CNF was smaller (2.0 mm). The EMW absorption performance is influenced by the impedance matching parameter (*M_Z_*) and the attenuation factor (*α*), which are calculated via Equations (3) and (4),^[^ ^45^, ^46^ ^]^

(3) $$M_{z}=\frac{2{Z^{\prime }}_{in}}{{\left|Z_{in}\right|}^{2}+1}$$

(4) $$\alpha =\frac{\sqrt{2}\pi f}{c}\;\sqrt{\left(\mu^{\prime \prime }\varepsilon^{\prime \prime }-\mu^{\prime }\varepsilon^{\prime }\right)+\sqrt{{\left(\mu^{\prime \prime }\varepsilon^{\prime \prime }-\mu^{\prime }\varepsilon^{\prime }\right)}^{2}+{\left(\mu^{\prime }\varepsilon^{\prime \prime }+\mu^{\prime \prime }\varepsilon^{\prime }\right)}^{2}}}$$

where *Z'_in_* is the real part input impedance. Typically, *M_z_* approaches 1, indicating that perfect impedance matching is achieved. As shown in Figure S4a_1_–a_3__(_Supporting Information), the impedance matching of the CNFs was the worst, which was probably due to the formation of a conductive network. Conversely, both ex‐Co‐CNF and in‐Co‐CNF exhibited good impedance matching, resulting from the introduction of a magnetic loss component. Moreover, in‐Co‐CNF possessed better impedance‐matching characteristics that could guarantee the absorption of incident EMW. Additionally, the largest *α* was achieved, as shown in Figure S5 (Supporting Information), contributing to the remarkable EMW absorption performance of the in‐Co‐CNF.

**Figure 5:**
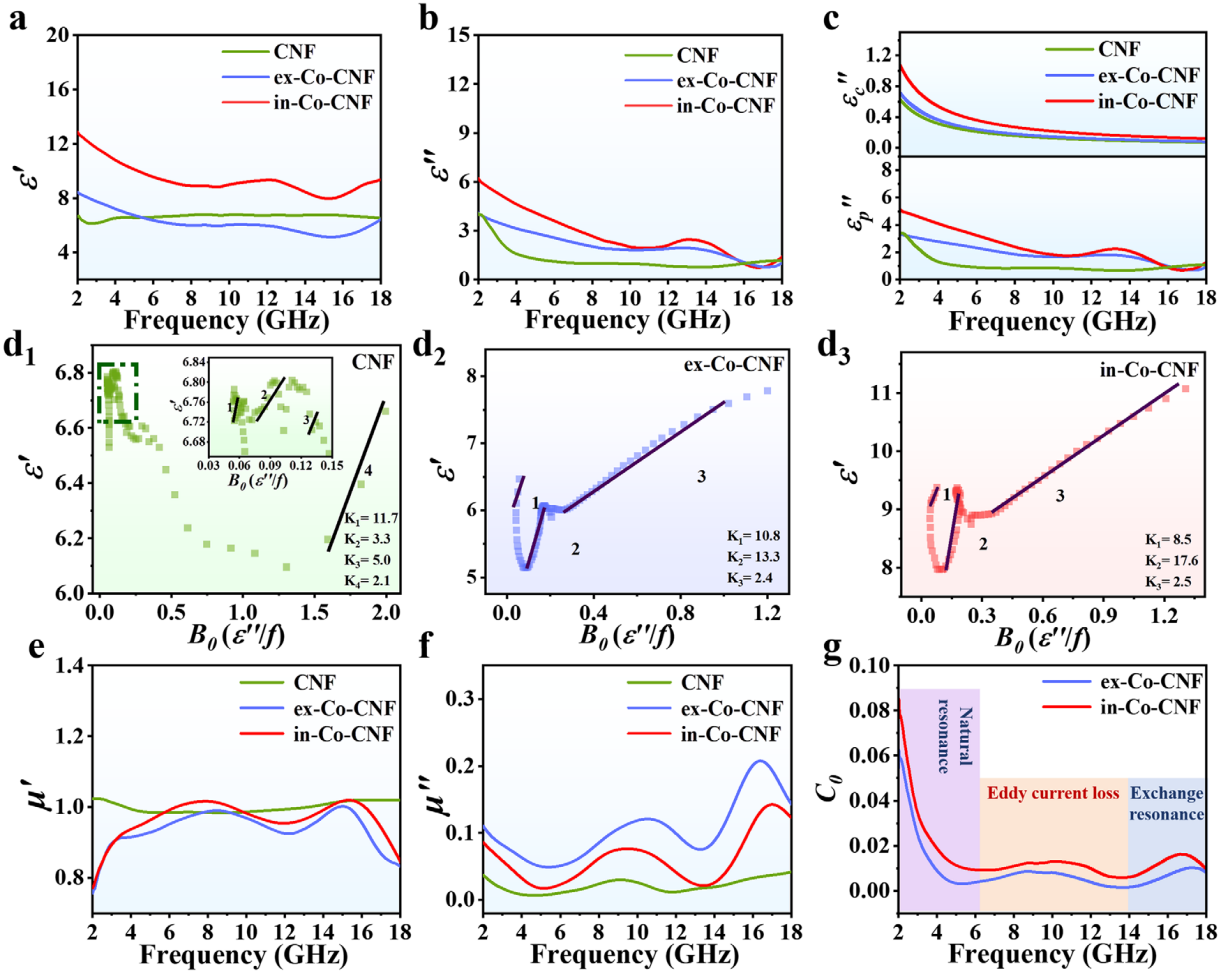
a) $\varepsilon^{\prime }$, b) $\varepsilon^{\prime \prime }$, and c) $\varepsilon_{c}^{\prime \prime }$ and $\varepsilon_{p}^{\prime \prime }$ of CNF, ex‐Co‐CNF, and in‐Co‐CNF; plot of $\varepsilon^{\prime }$ versus *B*_0_ of d_1_) CNF, d_2_) ex‐Co‐CNF, and d_3_) in‐Co‐CNF; e) μ′ and f) μ″ of CNF, ex‐Co‐CNF, and in‐Co‐CNF; and g) *C*_0_ curves of ex‐Co‐CNF and in‐Co‐CNF.

The EM parameters were examined to explore the EMW absorption mechanisms.^[^ ^47^ ^]^ As shown in **Figure** **5**a,b, both ex‐Co‐CNF and in‐Co‐CNF possessed higher values of $\varepsilon^{\prime }$ and $\varepsilon^{\prime \prime }$ compared to CNF. This might be due to the introduction of Co nanoparticles, which induced interfacial polarization and dipole polarization. Moreover, the $\varepsilon^{\prime }$ and $\varepsilon^{\prime \prime }$ of in‐Co‐CNF were greater than those of ex‐Co‐CNF. The higher values were attributed to the uniform distribution of small Co nanoparticles, which produced an increased number of polarization centers. Generally, $\varepsilon^{\prime \prime }$ can be calculated via Equations (5) and (6).^[^ ^48^ ^]^

(5) $$\varepsilon^{\prime \prime }=\varepsilon_{c}^{\prime \prime }+\varepsilon_{p}^{\prime \prime }$$

(6) $$\varepsilon_{c}^{\prime \prime }=\frac{\sigma }{2\pi f\varepsilon_{0}}$$

where $\varepsilon_{0}$ and *σ* represent the vacuum permittivity and electrical conductivity, respectively. $\varepsilon_{c}^{\prime \prime }$ and $\varepsilon_{p}^{\prime \prime }$ represent the conduction loss and polarization loss, respectively. It is evident from Figure S3 (Supporting Information) that in‐Co‐CNF had the highest *σ* of 0.12 S m^−1^. The difference in electronegativity between C and Co may induce charge rearrangement, promoting the electron transfer from Co to C.^[^ ^49^ ^]^ When the small‐sized Co nanoparticles were decorated on the surface of CNF, Co not only improved the electron movement but also reduced the interfacial contact resistance between the CNFs, thus leading to an increase in *σ*. In addition, the small‐sized Co nanoparticles provided more heterinterfaces, increasing the degree of interfacial polarization loss. Therefore, the $\varepsilon_{c}^{\prime \prime }$ and $\varepsilon_{p}^{\prime \prime }$ of in‐Co‐CNF were greater than those of ex‐Co‐CNF, as shown in Figure 5c. The Debye model was used to study the polarization loss in detail. The Cole‒Cole curves were plotted via Equations (7)–(9)^[^ ^50^, ^51^, ^52^ ^]^

(7) $${\left(\varepsilon^{\prime }-\frac{\varepsilon_{s}+\varepsilon_{\infty }}{2}\right)}^{2}+{\left(\varepsilon^{\prime \prime }\right)}^{2}={\left(\frac{\varepsilon_{s}-\varepsilon_{\infty }}{2}\right)}^{2}$$

(8) $$\varepsilon^{\prime }=\varepsilon_{\infty }+\frac{\varepsilon_{s}-\varepsilon_{\infty }}{1+{\left(2\pi f\right)}^{2}\tau^{2}}$$

(9) $$\varepsilon^{\prime \prime }=\frac{\omega \tau \left(\varepsilon_{s}-\varepsilon_{\infty }\right)}{1+{\left(2\pi f\right)}^{2}\tau^{2}}$$

where ɛ_∞_, ɛ_*s*_, and τ denote the optical permittivity, static permittivity, and relaxation time, respectively. As shown in Figure S6a (Supporting Information), the Cole‒Cole curve of the CNFs was composed almost entirely of arcs with no linear tail regions, indicating that the CNFs primarily exhibited polarization relaxation. This observation was similar to the results from the work of Sun and coworkers.^[^ ^53^ ^]^ In addition to the arcs, the Cole‒Cole curves of ex‐Co‐CNF and in‐Co‐CNF also exhibited linear tail regions, which are indicative of conductive loss (Figure S6b,c, Supporting Information).^[^ ^54^, ^55^, ^56^ ^]^ Equations (10) and (11) were used to deeply study the polarization relaxation in detail,^[^ ^57^ ^]^

(10) $$\varepsilon^{\prime }=B_{0}\;/2\pi \tau +\varepsilon_{\infty }$$

(11) $$\;B_{0}=\varepsilon^{\prime \prime }/f$$

According to Equations (10) and (11), a linear relationship between *B*_0_ and $\varepsilon^{\prime }$ indicates the presence of a single polarization process. As shown in Figure 5d_1_‒d_3_, multiple straight lines were obtained through linear fitting, implying the occurrence of multiple polarization relaxation processes. The N doping and oxygen vacancy defects within the carbon structure cause dipole polarization loss, whereas the heterointerfaces between Co and CNF contribute to interfacial polarization loss.

The theoretical density functional theory (DFT) calculations were conducted to further investigate the presence of heterogeneous atom‐induced interfacial polarization losses. Figure S7a (Supporting Information) shows the models and charge density images of Co and C. The differential charge density mapping of the Co─C is shown in Figure S7b (Supporting Information), where the green and yellow regions represent the areas of charge accumulation and charge depletion, respectively. At the interface between C and Co, electron transfer occurred because of their different electronegativities.^[^ ^49^ ^]^ C acted as an electron‐accepting layer, leading to negative charge accumulation. The Co side experienced electron loss, resulting in the formation of a positive charge region. Negative charge accumulation on one side of the interface and positive charge accumulation on the other side resulted in interfacial polarization loss. The total density of state (TDOS) is plotted in Figure S7c (Supporting Information). It is found that the C‒Co exhibited a higher TDOS near the Fermi level than C did, further indicating that the accumulation of charge at the heterointerface generated interfacial polarization loss.^[^ ^58^ ^]^

The complex permeability curves are shown in Figure 5e,f. The μ′ of the in‐Co‐CNF was greater than that of the ex‐Co‐CNF. This is due to the uniform dispersion of Co nanoparticles on the CNFs, which contributed to the high magnetic storage capacity. Conversely, in‐Co‐CNF possessed lower $\mu^{\prime \prime }$ than ex‐Co‐CNF did, which was attributed to the fact that the uniformly dispersed small‐sized Co nanoparticles induced a magnetic field to resist the original magnetic field, thereby suppressing magnetic loss.^[^ ^59^ ^]^ The magnetic loss mechanism was explored in greater detail via the *C_0_* value, as shown below^[^ ^60^, ^61^ ^]^

(12) $$C_{0}=\mu^{\prime \prime }\;{\left(\mu^{\prime }\right)}^{-2}f^{-1}$$

In the frequency‐dependent *C_0_* curve, natural resonance loss typically dominates the lower frequency range, whereas exchange resonance loss is observed in the higher frequency region.^[^ ^47^, ^62^ ^]^ The *C_0_* value does not vary with frequency when eddy current loss dominates as the primary mechanism for magnetic loss.^[^ ^63^, ^64^ ^]^ As shown in Figure 5g, the magnetic loss of ex‐Co‐CNF and in‐Co‐CNF was attributed to natural resonance (2.0‒6.0 GHz), eddy current loss (6.0‒14.0 GHz) and exchange resonance (14.0‒18.0 GHz). In the low‐frequency region, natural resonance originates from the intrinsic spin dynamics of the Co nanoparticles. Compared with those in ex‐Co‐CNF, the smaller and uniformly distributed Co nanoparticles in in‐Co‐CNF promoted a more pronounced natural resonance. In the middle and high‐frequency regions, the improved dispersion of Co nanoparticles in the in‐Co‐CNF facilitated the generation of eddy currents and exchange resonance losses. Consequently, greater magnetic loss of in‐Co‐CNF was observed, which in turn contributed to the enhanced EMW absorption performance.

**Figure 6:**
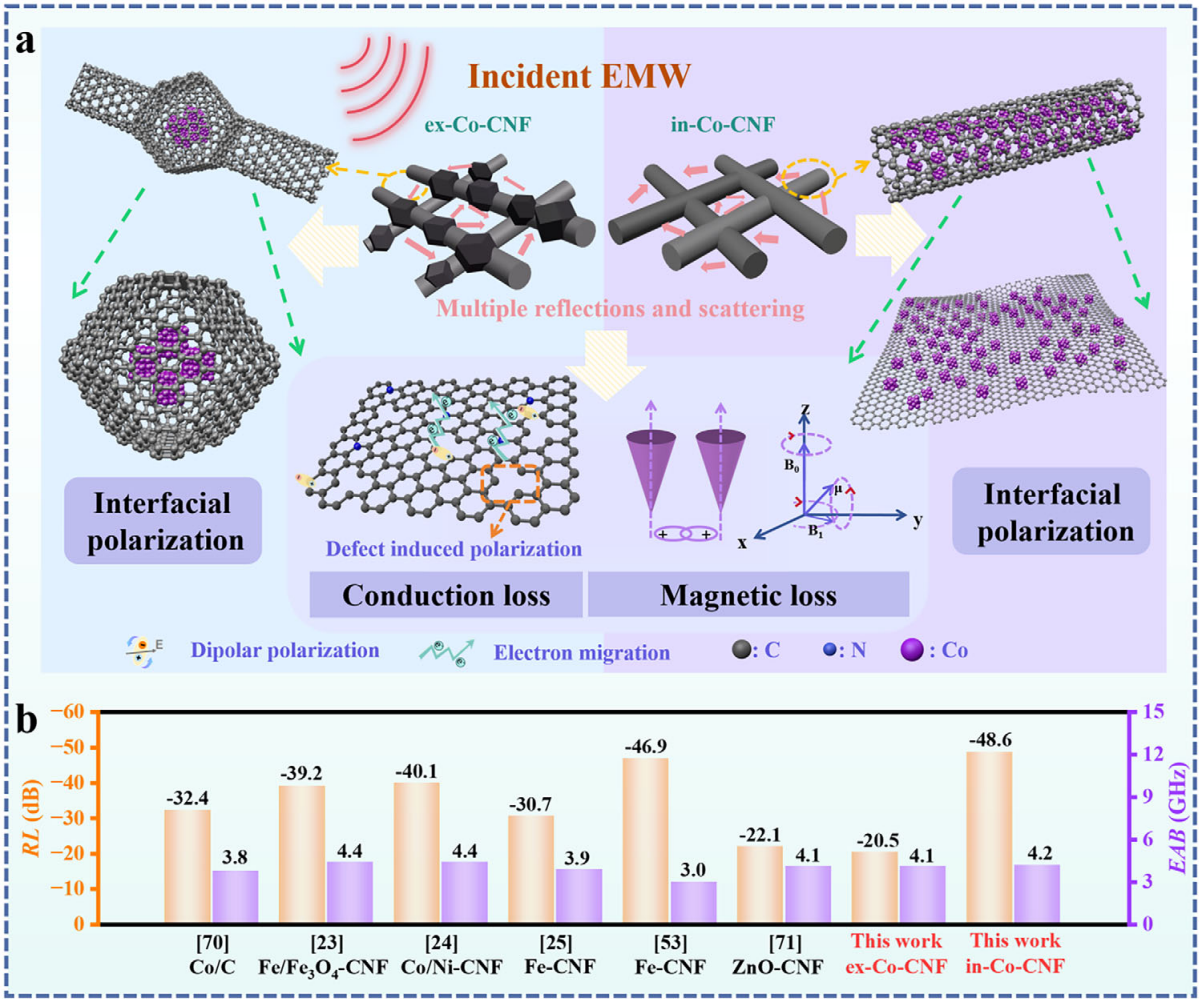
a) Representation of EMW absorption mechanisms of ex‐Co‐CNF and in‐Co‐CNF; b) comparison of in‐Co‐CNF with other similar EMW absorbing materials.

**Figure** **6**a shows the EMW absorption mechanisms of ex‐Co‐CNF and in‐Co‐CNF prepared via ex situ and in situ electrospinning strategies. The overlaps of the nanofibers provided a porous network, facilitating multiple reflections and scattering of the EMW. The CNFs created conductive networks,^[^ ^65^ ^]^ resulting in conduction loss. The presence of oxygen vacancies, N doping, and defects in the carbon structures contributed to dipole polarization.^[^ ^66^ ^]^ Because the Co contents in both ex‐Co‐CNF and in‐Co‐CNF were nearly the same, the size and distribution of Co nanoparticles played vital roles in the EMW absorption performance. The large‐sized Co nanoparticles were located at the framework of the carbon skeleton derived from ZIF‐67 in ex‐Co‐CNF. The introduction of Co nanoparticles created a heterointerface between Co and the carbon structure, resulting in charge accumulation, which promoted interfacial polarization. Furthermore, the introduction of Co nanoparticles induced considerable magnetic loss. In contrast, the collapse of the ZIF‐67 framework in the in‐Co‐CNF resulted in the uniform dispersion of small Co nanoparticles in the carbon network, creating more heterointerfaces and thereby enriching the polarization centers.^[^ ^67^ ^]^ Moreover, the small Co nanoparticles provided pronounced surface anisotropy, leading to improved magnetic loss.^[^ ^68^, ^69^ ^]^ In short, the unique characteristics of in‐Co‐CNF contributed to improved absorption capabilities owing to the combined effects of various EMW absorption mechanisms.

The EMW absorption properties of in‐Co‐CNF compared with those of other similar materials are summarized in Figure 6b. The EMW absorption properties were enhanced by designing carbon with magnetic metals or metal oxides. Qiang et al.^[^ ^70^ ^]^ prepared Co/C composites via direct carbonization of ZIF‐67. The obtained Co/C composites exhibited an *RL*_min_ of −32.4 dB and an *EAB* of 3.8 GHz. Fe/Fe_3_O_4_‐CNF,^[^ ^23^ ^]^ Co/Ni‐CNF,^[^ ^24^ ^]^ Fe‐CNF,^[^ ^25^, ^53^ ^]^ and ZnO‐CNF^[^ ^71^ ^]^ were prepared via the ex situ electrospinning method to construct MOF‐derived CNF composites. For example, Sun et al.^[^ ^53^ ^]^ prepared Fe‐CNF by direct electrospinning of a solution with pre‐synthesized Fe‐MOF nanoparticles. The *RL*_min_ of −46.9 dB and an *EAB* of 3.0 GHz were obtained. Figure 6b shows that the performance of in‐Co‐CNF, which exhibits an *RL*_min_ of −48.6 dB and an *EAB* of 4.2 GHz, is comparable to those of similar materials reported in the literature. However, cross‐study comparisons are often influenced by external factors, such as paraffin addition ratios or differences in precursor concentrations during preparation, which can significantly impact the results and reduce the accuracy of direct comparisons. In our own work, the comparison between ex‐Co‐CNF, which achieves an *RL*_min_ of −20.5 dB and an *EAB* of 4.1 GHz, and in‐Co‐CNF clearly demonstrates that the in situ electrospinning method is more advantageous for preparing high‐performance EMW absorption materials.

**Figure 7:**
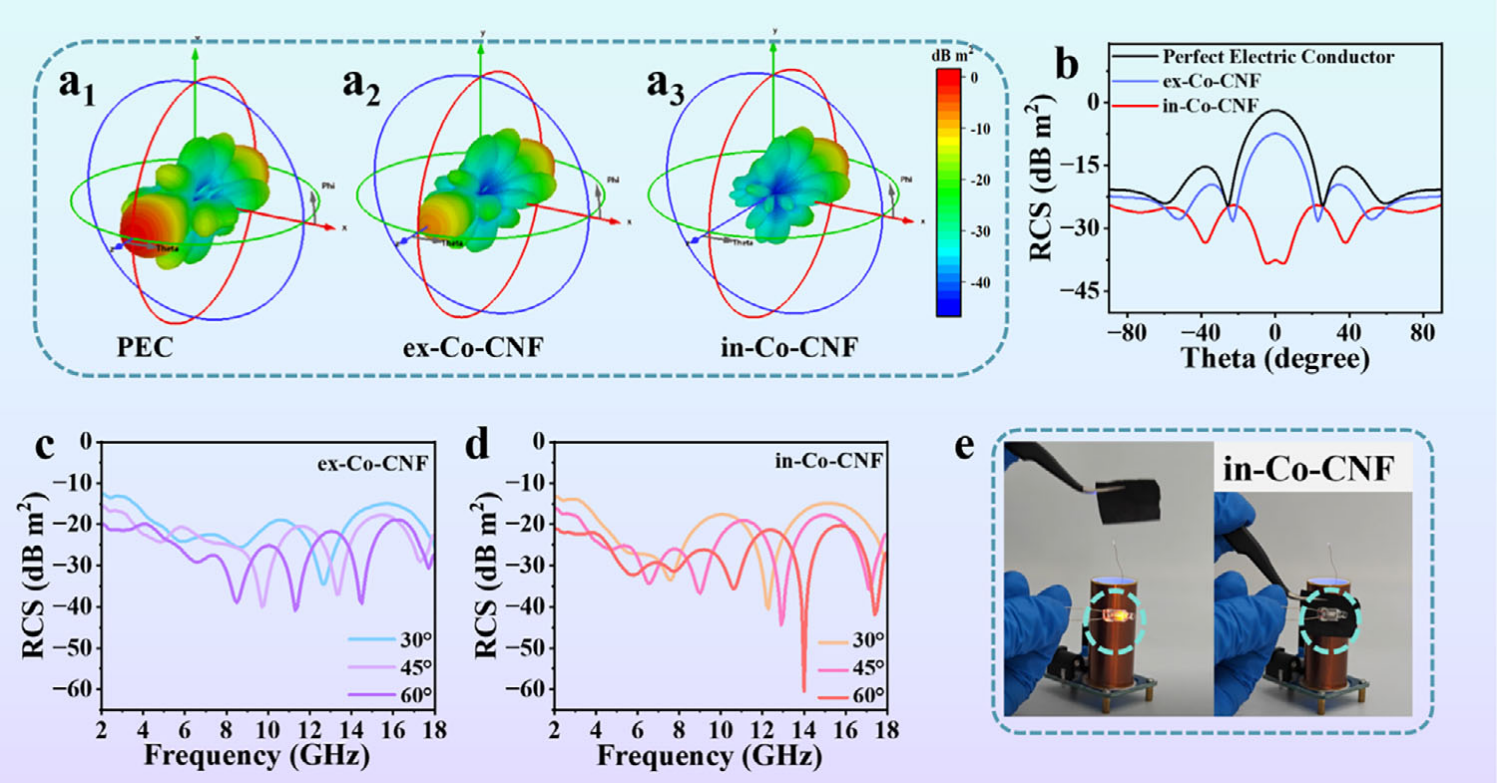
3D RCS of a_1_) PEC, PEC layer coated with a_2_) ex‐Co‐CNF, and a_3_) in‐Co‐CNF; b) 2D RCS of PEC and PEC layers coated with ex‐Co‐CNF and in‐Co‐CNF; RCS reduction of c) ex‐Co‐CNF and d) in‐Co‐CNF at selected scanning angles; e) EMW absorption of in‐Co‐CNF in Tesla wireless transmission.

To assess potential applications, radar cross‐section (RCS) simulations were conducted.^[^ ^72^ ^]^ Detailed information is available in the Supporting Information.Figure **7**a_1_‒a_3_ shows the 3D RCS simulation results at a fixed frequency of 6.8 GHz. A smaller radar scattering signal was observed for in‐Co‐CNFs than for ex‐Co‐CNFs. Figure 7b shows the 2D RCS simulation results of the sample in the range of −90‒90°. The RCS value of in‐Co‐CNF (−37.3 dB m^2^) was lower than that of ex‐Co‐CNF (−8.1 dB m^2^) at *θ* = 0°, indicating its outstanding EMW absorption capability. The RCS curves of ex‐Co‐CNF and in‐Co‐CNF were also plotted at selected angles of 30, 45, and 60°, as shown in Figure 7c,d. The in‐Co‐CNF clearly exhibited highly efficient EMW absorption at all three detection angles. Finally, the feasibility of using ex‐Co‐CNF and in‐Co‐CNF for EMW absorption was confirmed through Tesla wireless transmission experiments. The Tesla coil generates EMW that can ionize the surrounding air, resulting in lighting of the lamp. Interestingly, the lamp did not work after the in‐Co‐CNF film was inserted (Figure 7e) because the EMW produced by the Tesla coil was absorbed by the film. A demonstration of ex‐Co‐CNF and paper is also shown in Figure S8 and Video S1 (Supporting Information) It is clear that the surrounding EMW can be blocked by ex‐Co‐CNF, whereas inserting a paper did not work. The results clearly revealed that both ex‐Co‐CNF and in‐Co‐CNF with thin thicknesses have potential applications in EMW absorption.

## 3 Conclusion

In conclusion, this work compared EMW‐absorbing Co‐CNF composites fabricated via ex situ and in situ electrospinning strategies. The unique transformation mechanism from ZIF‐67 to Co nanoparticles was explored. The Co nanoparticles derived from ZIF‐67 regulated the EM parameters, which optimized the impedance matching and improved the attenuation coefficient. A uniform distribution of small‐sized Co nanoparticles at the surface of in‐Co‐CNF prepared via an in situ electrospinning strategy was attained, whereas ex‐Co‐CNF prepared via an ex situ electrospinning strategy possessed large Co nanoparticles that were encapsulated in the framework of the carbon skeleton derived from ZIF‐67. The small size of the Co nanoparticles resulted in a large specific surface area, which enhanced both dipole polarization and interfacial polarization. Moreover, the presence of uniformly distributed Co nanoparticles improved the conduction loss and magnetic loss. Consequently, in‐Co‐CNF demonstrated remarkable EMW absorption performance, with an *RL*_min_ of −48.6 dB (3.5 mm, 6.8 GHz) and an *EAB* of 4.2 GHz. Additionally, RCS simulations and Tesla wireless transmission experiments validated the highly efficient EMW absorption capability of in‐Co‐CNF. This work is anticipated to offer valuable guidance for the design of new‐generation fibrous EMW‐absorbing materials.

## 4 Experimental Section

### Materials

Bis(acetylacetonato) cobalt (C_10_H_16_CoO_4_, 99%) was acquired from Beijing InnoChem Science & Technology Co., Ltd. 2‐Methylimidazole (C_4_H_6_N_2_) and cobalt(II) nitrate hexahydrate (Co(NO_3_)_2_·6H_2_O, analytical reagent, 99%) were obtained from Aladdin Chemical Reagent Co., Ltd. Polyacrylonitrile (PAN) was acquired from Bide Pharmatech Co., Ltd. Ethanol (C_2_H_5_OH, AR, ≥99.7%) was obtained from Sinopharm Chemical Reagent Co., Ltd. N,N‐dimethylformamide (DMF, analytical reagent) was obtained from Meryer Biochemical Technology Co., Ltd.

### Preparation of Ex‐Co‐CNF by an Ex situ Electrospinning Process

A total of 1.19 g Co(NO_3_)_2_·6H_2_O and 1.69 g 2‐MI were dissolved in 52.5 mL C_2_H_5_OH and 17.25 mL C_2_H_5_OH, respectively, to obtain solutions A and B. After mixing solutions A and B, the mixture was magnetically stirred for 24 h. The precipitates were obtained by centrifugation at 9000 rpm for 12 min. After being washed, the ZIF‐67 nanoparticles were obtained by freeze‐drying for 6 h. To achieve a homogeneous solution, 0.9 g ZIF‐67, and 0.71 g PAN were ultrasonically dispersed in 5.68 g DMF and then magnetically stirred for 3 h at 65 °C. A voltage of 18 kV and a feeding rate of 0.6 mL h^−1^ were applied for the electrospinning process. The obtained samples were then subjected to thermal oxidation at 250 °C for 2 h. Afterward, the samples were heated at 800 °C for 2 h in an inert gas (Ar). Finally, ex situ synthesized Co‐C nanofibers (ex‐Co‐CNF) were obtained.

### Preparation of In‐Co‐CNF by an In situ Electrospinning Process

PAN (1.0 g) was mixed with 8.0 g DMF by magnetic stirring for 4 hours to prepare a PAN solution. Afterward, 1.5 g bis(acetylacetonato) cobalt was transferred to the PAN solution, followed by stirring for 6 h to form a solution. The same parameters were controlled during the electrospinning process as described above. The obtained Co^2+^/PAN nanofibers were dried at 50 °C for 12 h. The electrospinning precursor solution was prepared by dissolving 1 g 2‐MI in 100 mL. Afterward, all the as‐spun fibers were immersed. During this process, Co^2+^ coordinated with 2‐MI, resulting in the in situ synthesis of ZIF‐67 crystals on the PAN nanofibers (as shown in Figure 1). After 2 h, ethanol was used to wash the composite nanofibers. After being freeze‐dried for 6 h, the obtained nanofibers were subjected to heat treatment as described above. Finally, the in situ synthesized Co/C nanofibers (in‐Co‐CNF) were obtained.

### Characterization

The morphology, microstructure, and composition of the composite nanofibers were characterized through FESEM (Regulus 8230) and TEM (FEI Tecnai F20) with energy dispersive X‐ray (EDX) spectroscopy. The crystal structure and composition were characterized via X‐ray diffraction (XRD, Bruck D2 PHASER), X‐ray photoelectron spectroscopy (XPS, Phoibos 100 spectrometer), and Raman spectroscopy (Renishaw in Va). The Co content of the samples was measured by Inductively Coupled Plasma (ICP, Leeman Labs Prodigy 7). The BET surface area was measured under nitrogen atmosphere conditions via a microparticle analyzer (Microparticle ASAP 2460). The electrical conductivity was measured via the two‐probe method (KDY‐2). To prepare the samples for EMW absorption tests, 20 wt.% nanofibers were homogeneously dispersed in paraffin. The EM parameters were characterized via a vector network analyzer (VNA, ZNB‐20). The theoretical DFT calculations were performed via the Vienna Ab initio Simulation Package (VASP, 6.3.2). The RCS was obtained via the software CST Microwave Studio 2018. The simulation details are available in the Supporting Information.

## Conflict of Interest

The authors declare no conflict of interest.

## Supporting information



Supporting Information

Supporting Information Video S1

## Data Availability

The data that support the findings of this study are available from the corresponding author upon reasonable request.
