# Supplementary material for: Mechanistic Insights into the Structural Evolution of ZIF‐67 via Electrospinning Strategy Toward High Electromagnetic Wave Absorption Performance of ZIF‐67‐Derived Carbon Nanofibers
Source: Adv Sci (Weinh). 2025 Apr 7;12(26):2502560. doi: 10.1002/advs.202502560 (PMC12244997; doi:10.1002/advs.202502560)
Supplement: Supplementary file 1 — Supporting Information [file ADVS-12-2502560-s001.docx]

Supporting Information

**Mechanistic Insights into the Structural Evolution of ZIF-67 via Electrospinning Strategy Toward High Electromagnetic Wave Absorption Performance of ZIF-67-Derived Carbon Nanofibers**

*Xinhui Cao, Xinyi Wu, Xue Wang,* *Jiamei Luo, Zhe Zhang,* *Yi Xue, Guoliang Zhang, Liying Zhang*, Hui Zhang*, Jianyong Yu*


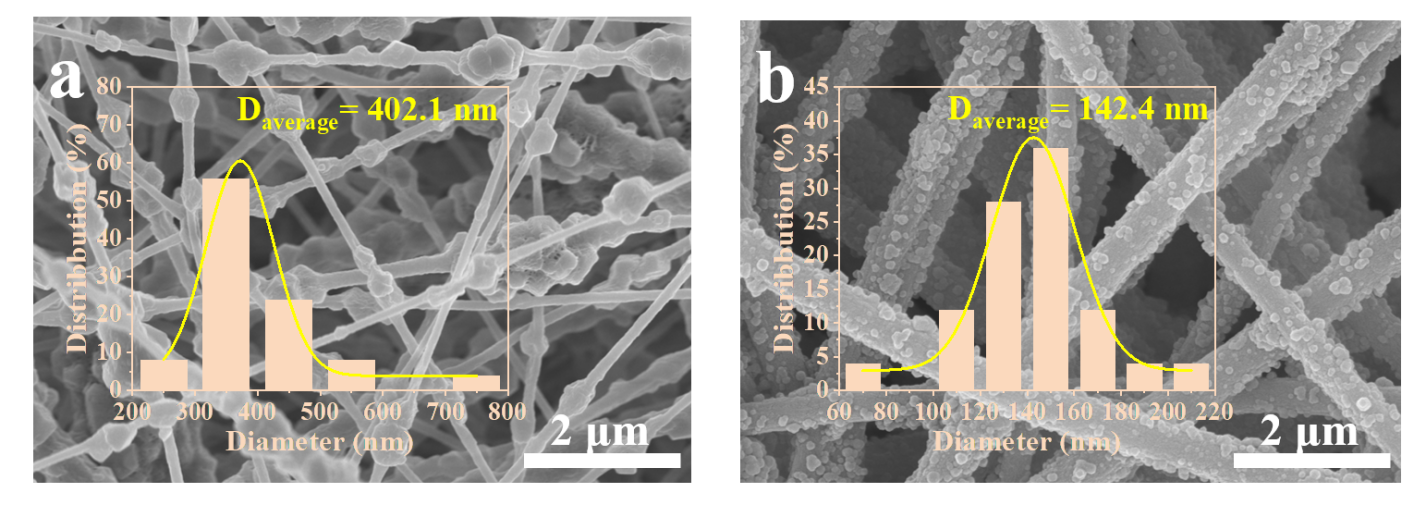


**Figure S1**. Particle size distributions of the ZIF-67 nanoparticles in a) ex-ZIF-67-PAN and b) in-ZIF-67-PAN.


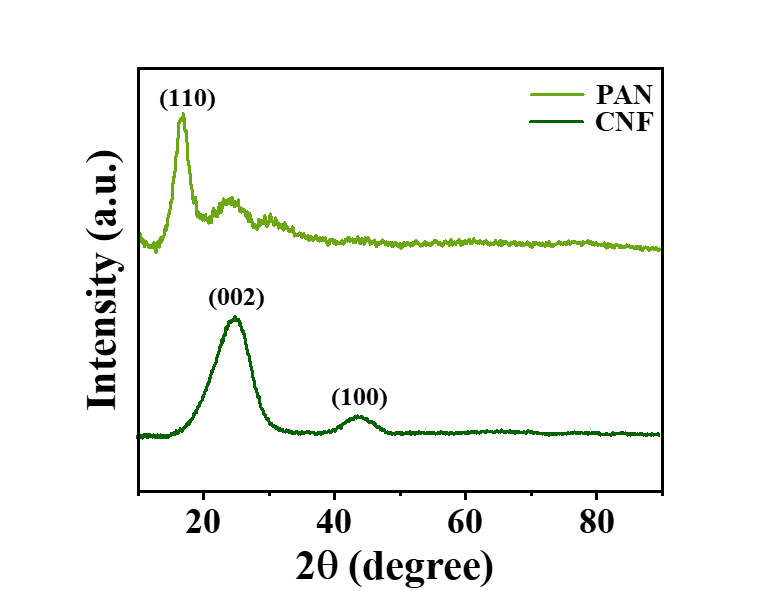


**Figure S2**. XRD diffraction patterns of PAN and CNF.


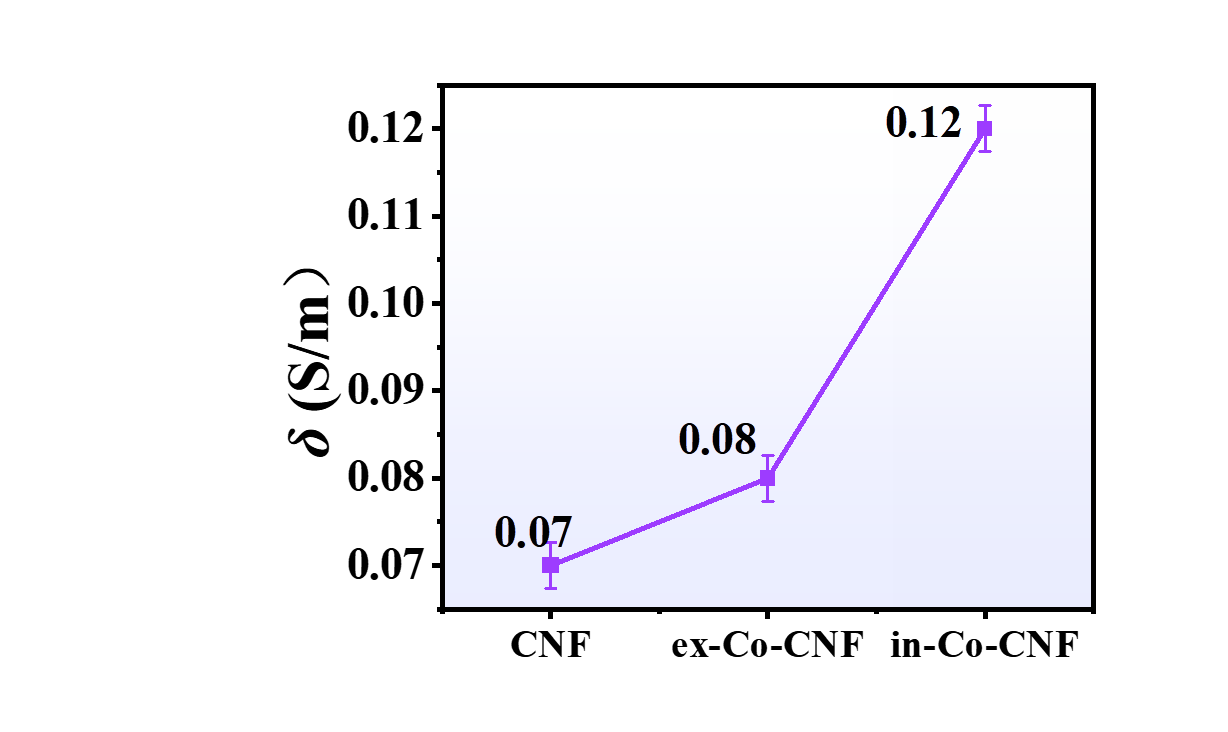


**Figure S3**. Electrical conductivity of CNF, ex-Co-CNF, and in-Co-CNF.


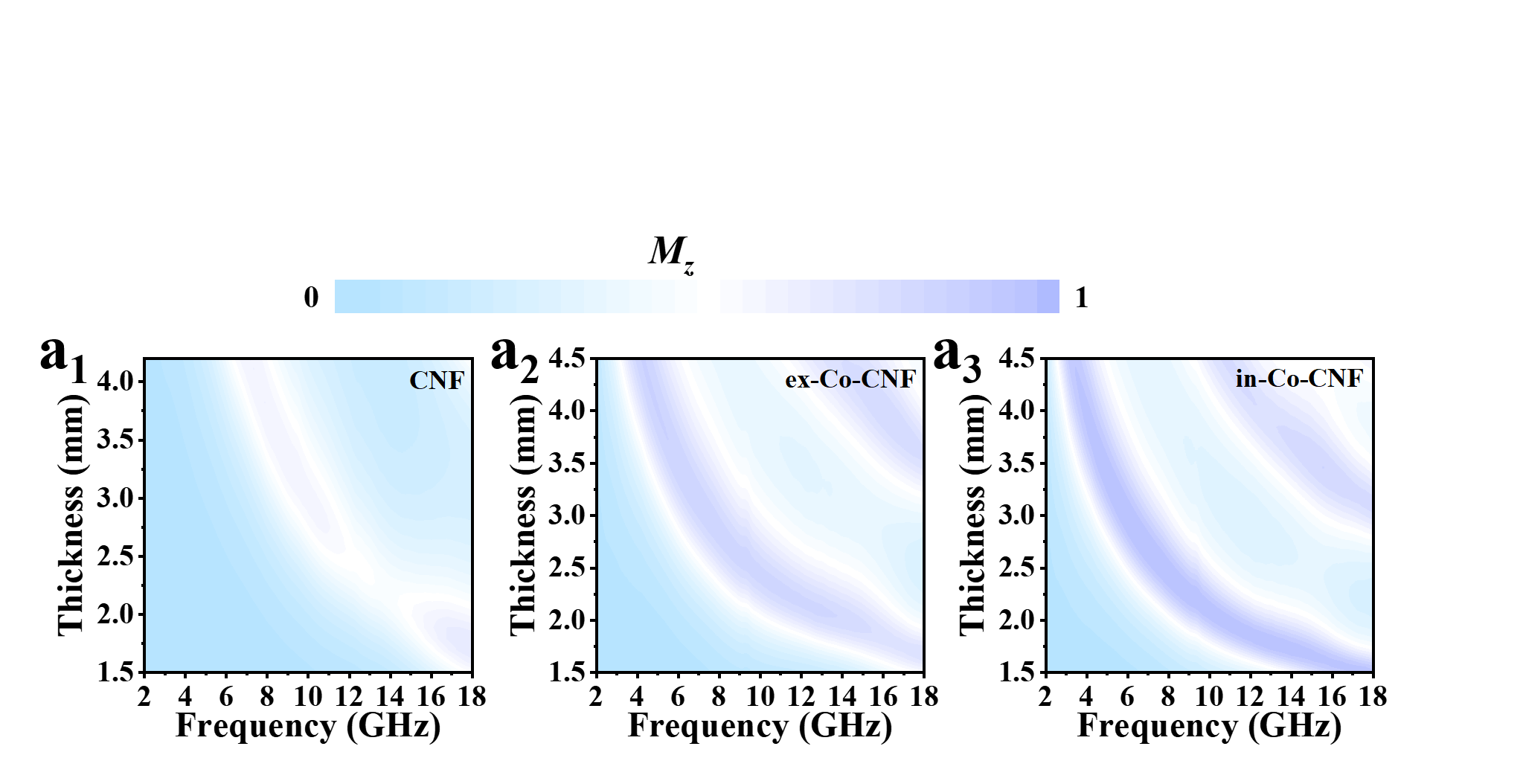


**Figure S4**. *M_z_* values of a_1_) CNF, a_2_) ex-Co-CNF, and a_3_) in-Co-CNF.

**
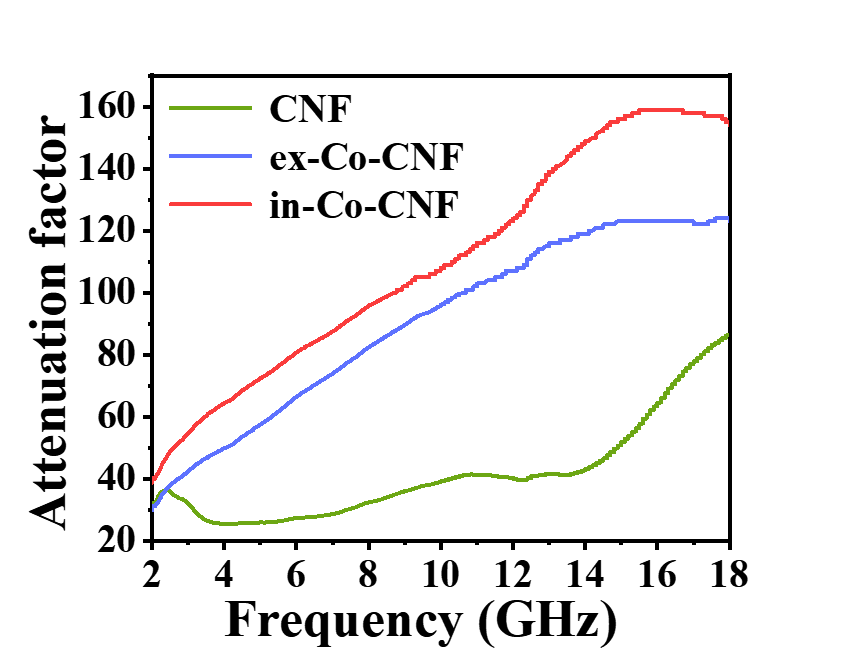
**

**Figure S5**. Attenuation coefficients of CNF, ex-Co-CNF, and in-Co-CNF.


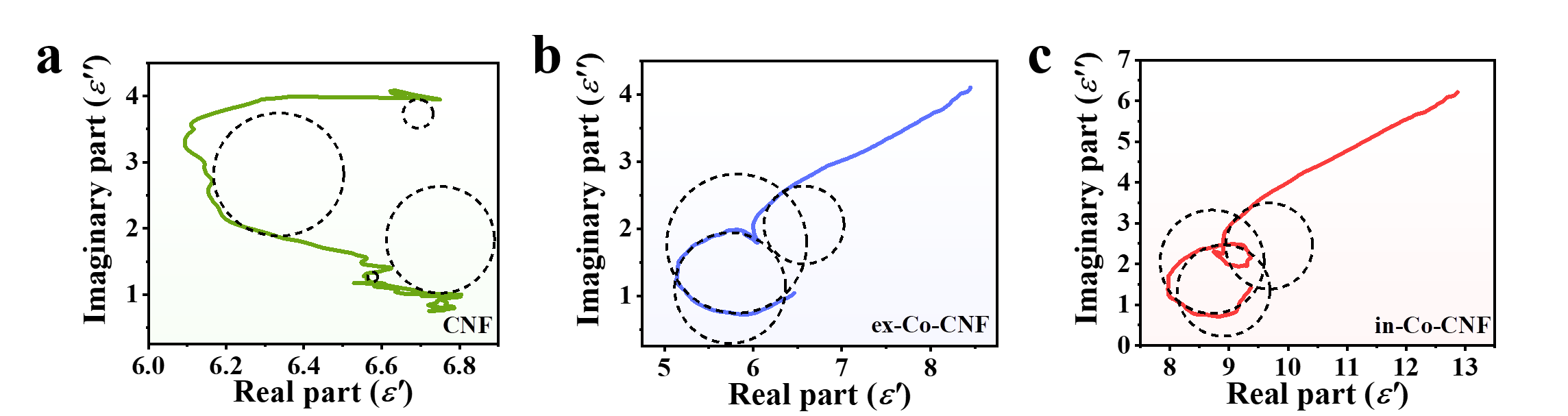


**Figure S6**. Cole‒Cole semicircles of a) CNF, b) ex-Co-CNF, and c) in-Co-CNF.


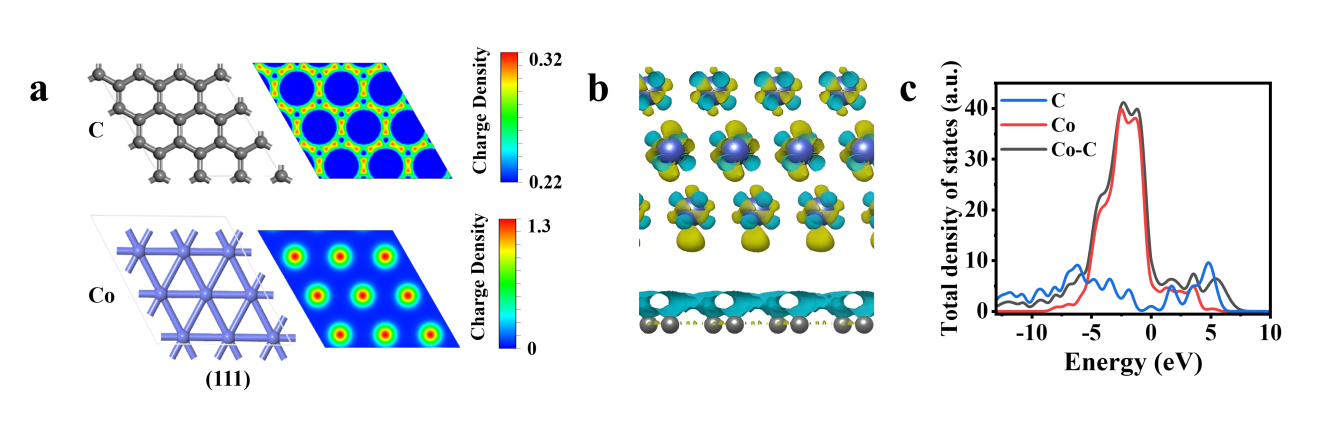


**Figure S7**. a) Simulation models and the charge density images of Co and C; b) differential charge density mapping of the Co-C heterointerface; c) total density of states of C, Co, and Co-C.


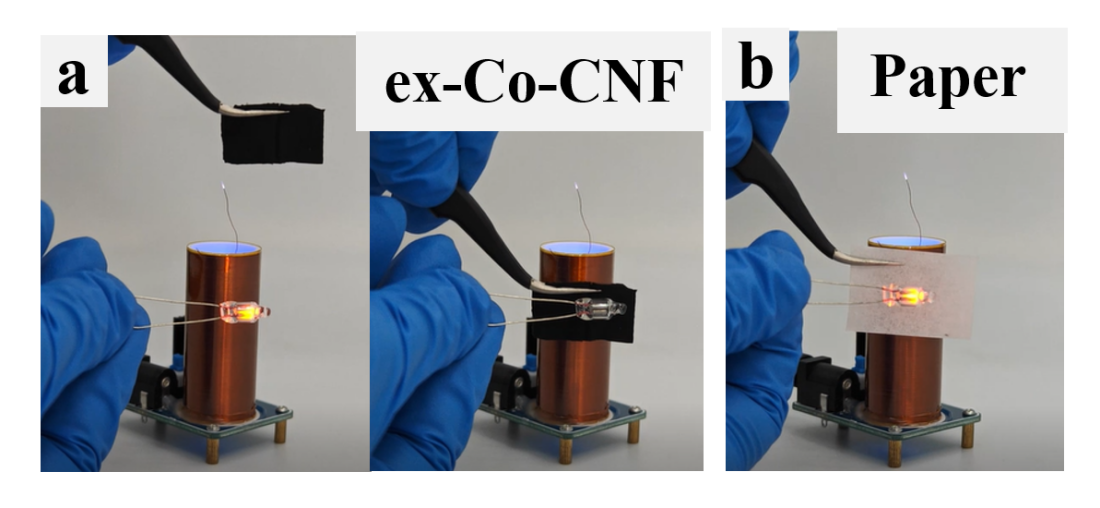


**Figure S8**. Demonstration of EMW absorption of a) ex-Co-CNF and b) paper in Tesla wireless transmission.

**Density Functional Theory (DFT) Calculations**

The VASP (version 6.3.2) was used to perform the DFT calculations. The exchange-correlation potential was modelled using the Perdew-Burke-Ernzerhof (PBE) functional within the Generalized Gradient Approximation (GGA). The Projector Augmented-Wave (PAW) pseudopotentials described the core electrons, and a plane-wave basis set was employed with a kinetic energy cut-off of 400 eV for the valence electrons. The van der Waals interactions between the adsorbate and the surface were treated via Grimme’s zero-damping DFT-D3 method. Structural relaxations were carried out until the forces on each ion were less than 0.05 eV/Å, and the energy convergence criterion was set to 10^-5^ eV. Sampling of the Brillouin zone was conducted via a Monkhorst-Pack k-point mesh with a grid size of 3 × 3 × 1.

**Radar cross section (RCS) simulation**

The CST was used for simulation to derive RCS values for the far-field response. The square model comprised a lower perfect electrically conductive (PEC) layer (thickness = 2.0 mm) and an upper absorber layer (thickness = 3.5 mm) with 100 mm sides. The monostatic RCS was determined through calculations performed via a time domain solver. The simulation model is positioned on the XOY plane, with the EM wave propagating along the negative direction of the Z-axis. The electric polarization direction of the EM wave was deliberately set to propagate along the X-axis. Open boundary conditions were implemented in all directions, whereas the scattering direction was defined by theta and phi in polar coordinates. The RCS can be defined by Equation S1:

$\sigma(dB m^{2})=10 lg \left( \frac{4\pi S}{\lambda^{2}}\times\left| \frac{E_{s}}{E_{i}} \right| \right)^{2}$ (S1)

*S* signifies the area of the model, *λ* represents the wavelength, and *E_s_* and *E_i_* denote the electric field intensities of the transmitting and receiving EM waves, respectively.

**Appendix**

**1.** **Calculation of the number of cobalt atoms in a Co nanoparticle**

*ρ*= $\frac{m}{V}$ or *ρ*= $\frac{\frac{m}{M}}{V}$ (S2)

where *ρ* represents density, *m* represents mass, *M* represents relative atomic mass, and *V* represents volume. The density and relative atomic mass of Co are 8.9 g/cm^3^ and 58.933 g/mol, respectively. According to Equation S2, the density of Co can be converted to another expression: *ρ* = 1.508×10^-22^ mol/nm^3^.

On the basis of the TEM images in **Figure 2**d_2_ and f_2_, approximately 39 and 12 nm Co nanoparticles were obtained in ex-Co-CNF and in-Co-CNF, respectively. The following equations can be used for calculating the number of Co atoms in a Co nanoparticle.

Volume: $V_{r}=\frac{4}{3}\pi r^{3}$ (S3)

Molar quantities：*n* = *ρV_r_* (S4)

Total number：*N_T_* = *n* × N_A_ (S5)

where *V_r_* is the volume of a sphere, *r* is the atomic radius, *n* is the molar mass, *N_T_* is the total number of atoms, and N_A_ is Avogadro's constant (6.02×10^23^). For 39 nm Co nanoparticles, $V_{r}=\frac{4}{3}\pi r^{3}=\frac{4}{3}\pi\left( \frac{39}{2} \right)^{3}=31059$ nm^3^, *n* = *ρV* = 1.508×10^-22^× 31043 = 4.68×10^-18^ mol, *N_T_* = *n* × N_A_ = 4.68×10^-18^ × 6.02 ×10^23^ = 2819618. Similarly, for a 12 nm Co nanoparticle, *N_T_* = 82137.

**2. Calculation of the total number of cobalt atoms in ZlF-67**

ZIF-67 exhibits cubic crystal symmetry with unit cell parameters of *a*=*b*=*c*=16.907 Å and *α*=*β*=*γ*=90°, and its cell volume (*V_c_*) is 4833.41 Å^3^.^[1]^ The number density (*ρ_n_*) of Co can be calculated via Equation S6:

$\rho_{n}=\frac{N_{c}}{V_{c}}$ (S6)

where *N_c_* denotes the number of atoms contained in a unit cell. On the basis of the unit cell structure of ZIF-67 (**Figure S**9), *N_c_* = 12, indicating that one-unit cell of ZIF-67 contains 12 Co atoms. Hence, $\rho_{n}=\frac{N_{c}}{V_{c}}=\frac{12}{4833.41}=2.48$ nm^-3^.


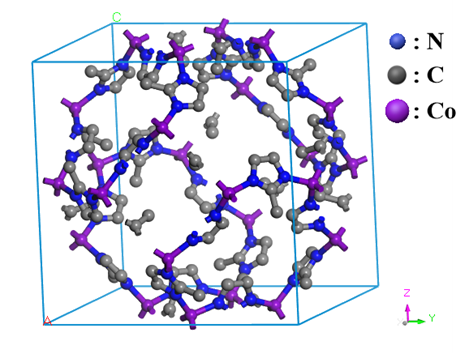


**Figure S9**. Unit cell of ZIF-67 (derived from Materials Studio Software)

The total number of Co atoms can be calculated via Equation S7 and S8:

$V_{rd}=\frac{16}{9}\sqrt{3}a^{3}$ = $\frac{16}{9}\sqrt{3}{(0.5b)}^{3}$ (S7)

*N_N_* = $\rho_{n}V_{rd}$ (S8)

where *V_rd_* is the volume of the rhombic dodecahedron, *a* is the side length of the rhombic dodecahedron, *b* is the particle size, which is equal to 2a, and *N_N_* is the number of Co atoms (**Figure S10**). **When the particle size of ZIF-67 is 402.1 nm**, $V_{rd}=\frac{16}{9}\sqrt{3}{(0.5b)}^{3}$=$\frac{16}{9}\sqrt{3}{(0.5\times402.1)}^{3}$=25023631 nm^3^ and *N_N_*=$\rho_{n}V_{rd}$=2.48×25023631=62058606. Thus, the number of 39 nm Co atoms in the 402.1 nm ZIF-67 particles is $\frac{N_{N}}{N_{T}}=\frac{62058606}{2819618}$ ≈ 22. Similarly, **when the particle size of ZIF-67 is 142.4 nm**, $V_{rd}=\frac{16}{9}\sqrt{3}{(0.5b)}^{3}$=$\frac{16}{9}\sqrt{3}{(0.5\times142.4)}^{3}$=1111420 nm^3^ and *N_N_*=$\rho_{n}V_{rd}$=2.48×1111420=2756322. Thus, the number of 12 nm Co atoms obtained from the 118.3 nm ZIF-67 particles is $\frac{N_{N}}{N_{T}}=\frac{2756322}{82137}$≈ 34.

The meaning of each letter is summarized in **Table S1** below.


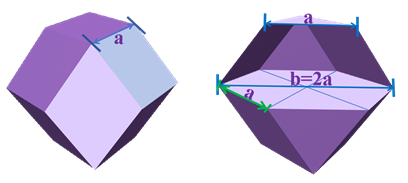


**Figure S10**. Rhombic dodecahedron of a ZIF-67 unit (*a* is the edge length and *b* is the particle size).

**Table S1. Co content in ex-Co-CNF and in-Co-CNF measured by ICP.**

| **Element** | **ex-Co-CNF** | **in-Co-CNF** |
| --- | --- | --- |
| **Co (mg/g)** | 242.46 | 244.35 |

**Table S2. The meaning of the alphabet**

| **Letter** | **Meaning of each letter** |
| --- | --- |
| *ρ* | Density |
| 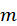 | Mass |
| *V* | Volume |
| *V_r_* | Volume of the sphere |
| 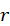 | Atomic radius |
| *n* | Molar mass |
| *N_T_* | Total number of atoms |
| N_A_ | Avogadro’s constant (6.02×10^23^) |
| *V_c_* | Cell volume |
| *ρ_n_* | Number density |
| *N_c_* | Number of atoms containing in a unit cell |
| *V_rd_* | Volume of rhombic dodecahedron |
| *a* | Side lengths of a rhombic dodecahedron |
| *b* | Particle size (*b*=*2a*) |
| *N_N_* | Number of Co atoms |

**Reference**

[1] H. T. Kwon, H. Jeong, A. S. Lee, H. S. An, J. S. Lee, *Journal of the American Chemical Society* **2015**, *137*, 12304.
